# Medical Reform and the Individual Patient

**Published:** 1918-08-03

**Authors:** 


### The Hospital

The Workers' Newspaper of Administrative Medicine and Institutional Life, Administration, National Insurance and Health.

No. 1678, Vol. LXIV. SATURDAY, AUGUST 3, 1918. PRICE TWOPENCE

### MEDICAL REFORM AND THE INDIVIDUAL PATIENT.

Proposals for the reform of the medical profession and schemes indicating the direction in which this should be attempted are just now very much in evidence. Scarcely a week passes without an address or an article appearing in one or other of our medical contemporaries, and evidently the question is regarded as one of urgent moment. Two considerations appear to be mainly responsible for this position. First, it is recognised that the establishment of a Ministry of Health in the near future is highly probable, and it is accepted that this is likely to bring in its train some new and organised relations between the profession on the one hand and the State on the other. Next is the certainty that the end of the war will bring back to civil life some thousands of practitioners and that a large proportion of these, finding their practices vanished, will have to make a new start in life. Very naturally the profession is concerned with the effect which these two events are likely to exercise both on the work and status of the individual practitioner and on the relations of medicine to the public. So far the layman has not had much to say on these issues. Yet he has a very large interest in them and in the end his voice is sure to be heard.

Programmes and proposals, whether emanating from doctors or from politicians, must in the last analysis stand on their defence before a large non-professional jury, and only if they meet the general interest and general convenience can they hope for a favourable verdict. It may be doubted whether all the reformers have kept this consideration fully in view. Just now the public is accustomed to be ordered and directed by officials and authorities. It submits to these under the pressure of a great national necessity. But, unless we greatly mistake the temper of the nation, submission will not be the mood of the country when external circumstances cease their compelling sway. Then, at least in our expectation, the individual citizen will assert himself, and short work will be made both of men and measures whose rule for the time being is suffered with comparative calm. It is with this development, as we see the position, that medical reformers must reckon.

Now with medical work and enterprise the public is concerned both in a general and in a particular sense. The first of these interests is the organisation of the methods by which epidemic or other widespread diseases can be controlled or suppressed, and here it may be freely allowed that governing authorities may expect a fairly free hand so long as their methods have or seem to have the justification of success. If disease is preventable, measures which appear likely to prevent it will certainly command general assent even though they inflict inconvenience or annoyance on a certain number of individuals. All such proposals and undertakings stand as part of our social politics and can be discussed in a detached and impersonal fashion by the sanitary reformer, with the certainty that his less alert fellow-citizens will accept without active protest a good deal of autocratic ordering. The end is a large one and the methods of obtaining it are highly technical. Hence our pastors and masters may count on the assent of the plain man to their proposals even though these mean some sacrifice of individual liberty.

But no such forbearance can, we think, be expected when it is proposed to regulate the personal relations of the individual citizen to the medical profession. The citizen who is a sick man or recognises that he may be a sick man thinks not unnaturally that here is a matter in which no one has so close a concern and interest as himself, and his inclination is to conclude that his own judgment and choice form the most secure guide both in the selection of an adviser and in the definition of the relation which this adviser shall hold to himself. He may be ready to listen to suggestions and to proposals from others, but hardly to the voice of an authority seeking to impose a prescribed polity. Here, at all events, the Englishman's house is his castle, and we venture to say that reformers who ignore this claim are reckoning without their host. One of the provisions of the National Insurance Act which has caused widespread resentment is that which renders it difficult for an insured person to change his medical adviser, and we feel sure that any aggravation of this policy will excite among the public a determined opposition.

We are sometimes told that practitioners who have seen service in the Army will return to civil life so enamoured of the benefits of an organised and controlled medical service that their votes and influence will be lent with enthusiasm to a scheme which sets up a parallel position in civilian practice. Whether this conviction is or is not well founded we do not pretend to say. But even should it be strictly accurate, we dispute the suggested inference that such a conclusion settles the question. There will return with the doctors tens of thousands of laymen and a proportion of these will have had personal experience of the working of an "organised and controlled medical service." It will be important to know what they think of such an arrangement, and as they will have votes their views cannot be left out of the calculation. They are certainly not likely to be touched by collections of statistics which show how epidemic diseases have been successfully defied, nor by recitals of pathological and bacteriological triumphs. Let all these be allowed as admirable in themselves and hailed as inspiring guides to preventive medicine. But the personal issue remains and it concerns the individual and the family. Perhaps the sick person ought to be influenced by high scientific considerations. But as a matter of fact he is not. He is influenced by the desire to get well and he thinks one help to this end is the advice of the doctor of his choice. He wants to see this doctor and perhaps later he wants to get rid of him. These are blunt prosaic facts. Yet they are deeply set in human nature, and the medical reformer who ignores them will find them in array against him.

